# Deacidification of Cranberry Juice Reduces Its Antibacterial Properties against Oral Streptococci but Preserves Barrier Function and Attenuates the Inflammatory Response of Oral Epithelial Cells

**DOI:** 10.3390/foods10071634

**Published:** 2021-07-15

**Authors:** Geneviève Pellerin, Laurent Bazinet, Daniel Grenier

**Affiliations:** 1 Institute of Nutrition and Functional Foods (INAF), Department of Food Sciences, Université Laval, Quebec City, QC G1V 0A6, Canada; genevieve.pellerin.3@ulaval.ca (G.P.); Laurent.bazinet@fsaa.ulaval.ca (L.B.); 2 Laboratoire de Transformation Alimentaire et Procédés ÉlectroMembranaires (LTAPEM, Laboratory of Food Processing and ElectroMembrane Processes), Université Laval, Quebec City, QC G1V 0A6, Canada; 3 Oral Ecology Research Group, Faculty of Dentistry, Université Laval, Quebec City, QC G1V 0A6, Canada

**Keywords:** oral streptococci, dental caries, epithelial barrier, electrodialysis, cranberry juice, antibacterial, organic acids, inflammation

## Abstract

Cranberry (*Vaccinium macrocarpon*) may be a potent natural adjuvant for the prevention of oral diseases due to its anti-adherence, anti-cariogenic, and anti-inflammatory properties. However, the high titrable acidity of cranberry juice (CJ) has been reported to cause gastrointestinal discomfort, leading consumers to restrict their intake of this beverage. Electrodialysis with a bipolar membrane (EDBM) can reduce the organic acid content of CJ while retaining the flavonoids associated with potential health benefits. This study aimed to assess how the deacidification of CJ by EDBM impacts the antibacterial properties of the beverage against cariogenic (*Streptococcus mutans*, *Streptococcus sobrinus*) and commensal (*Streptococcus gordonii*, *Streptococcus oralis*, *Streptococcus salivarius*) streptococci, and how it affects oral epithelial barrier function and inflammatory response in an in vitro model. The removal of organic acids from CJ (deacidification rate ≥42%) reduced the bactericidal activity of the beverage against planktonic *S. mutans* and *S. gordonii* after a 15-min exposure, whereas only the viability of *S. gordonii* was significantly impacted by CJ deacidification rate when the bacteria were embedded in a biofilm. Moreover, conditioning saliva-coated hydroxyapatite with undiluted CJ samples significantly lowered the adherence of *S. mutans*, *S. sobrinus*, and *S. oralis.* With respect to epithelial barrier function, exposure to CJ deacidified at a rate of ≥19% maintained the integrity of a keratinocyte monolayer over the course of 24 h compared to raw CJ, as assessed by the determination of transepithelial electrical resistance (TER) and fluorescein isothiocyanate-conjugated dextran paracellular transport. These results can be in part attributed to the inability of the deacidified CJ to disrupt two tight junction proteins, zonula occludens−1 and occludin, following exposure, unlike raw CJ. Deacidification of CJ impacted the secretion of IL-6, but not of IL-8, by oral epithelial cells. In conclusion, deacidification of CJ appears to provide benefits with respect to the maintenance of oral health.

## 1. Introduction

Over 700 bacterial species have been detected in the oral cavity. Although most pose no threat to the maintenance of oral health, a minority are recognized pathogens [1]. The development of a dental biofilm begins with the establishment of streptococcal species, including *Streptococcus mitis*, *Streptococcus oralis*, *Streptococcus sanguinis*, and *Streptococcus gordonii*. These early colonizers use a wide variety of surface adhesins to bind to proteinaceous constituents of the acquired salivary pellicle that covers the tooth enamel, then firmly adhere to and grow on the tooth surface [2,3,4,5]. Although most early colonizers are commensal, they can also serve as anchor points for secondary colonizers, some of which are oral pathogens [4]. Additionally, the accumulation of dental biofilm can disrupt the oral mucosa, which serves as an interface that protects the host from environmental stresses and bacterial invasions of host tissues [6]. The oral epithelium is also regularly challenged by dietary constituents that may contain damaging agents. Preserving the integrity of the oral mucosa by limiting the build-up of dental biofilm and by consuming low stress-inducing foods is crucial in order to prevent infections and slow the development of severe oral diseases.

Mutans streptococci, mainly *Streptococcus mutans* and *Streptococcus sobrinus*, are key pathogens involved in the onset of dental caries [7,8,9,10]. Their acidogenic and aciduric natures give them a competitive advantage in the development of carious lesions. Their ability to form insoluble glucans from dietary sucrose via glucosyltransferases (GTFs) allows them to bind strongly and secure their attachment to tooth surfaces [11]. These enzymes also support the formation of a compact exopolysaccharide (EPS) matrix that limits compound diffusion. Lactate, which is abundantly produced from sugar by mutans streptococci, accumulates locally, causing a pH drop and enamel demineralization [4,12].

The American cranberry (*Vaccinium macrocarpon*), which is mainly consumed in the form of cranberry juice (CJ) [13], is regarded as a promising functional food for the prevention of chronic diseases such as cancer, heart diseases, type 2 diabetes, and oral diseases [14,15,16,17]. More specifically, the phenolic compounds of this berry, which include A-type proanthocyanidins (PACs), possess many bioactivities that can hinder the development of dental caries. Both CJ and cranberry phenolic extracts have been described as effective inhibitors of GTFs in mutans streptococci [18,19,20,21]. The inhibition of GTFs prevents the development of cariogenic biofilms in vitro and in vivo [18,19,20,21]. Moreover, the reported ability of cranberry polyphenols to block bacterial coaggregation could hamper the accumulation of dental plaque [22]. Cranberry polyphenols also lower the aciduricity of *S. mutans* by inhibiting the proton-translocating F_1_F_0_-ATPase activity that allows *S. mutans* to maintain an active metabolism under acidic stress [18,19].

Electrodialysis with bipolar membrane (EDBM), an electromembrane process, has recently been successfully used at a semi-industrial scale for the deacidification of CJ without altering the phenolic profile of the juice [23]. Aside from the ecoefficiency of EDBM [24], the benefit of this process lies in the selective removal of citric acid (CA) and malic acid (MA) from CJ [23,25], which are responsible for the gastrointestinal discomfort reported when the raw beverage is consumed [26,27] and contribute to enamel demineralization [28,29]. For instance, a CJ deacidification rate (DR) of ≥37% reached by EDBM, which translates into a ≥37% reduction in titratable acidity, significantly preserves the integrity of an intestinal epithelial barrier in an in vitro model [30]. This has been mainly associated with the removal of CA [31].

The present study investigated how the deacidification rate of CJ obtained by EDBM affects the potential benefits of the juice regarding the prevention of dental caries and the maintenance of the oral epithelial barrier. The specific objectives of the present study were to investigate how EDBM deacidification of CJ impacts (1) antibacterial activities against oral streptococci, (2) the barrier function of oral epithelial cells, and (3) the inflammatory response of oral epithelial cells.

## 2. Materials and Methods

### 2.1. Cranberry Juice

Pasteurized and clarified CJ produced from fresh fruits was obtained from Fruit d’Or (Plessisville, QC, Canada). The cranberry juice was kept frozen at −30 °C, and was thawed at 4 °C before deacidification.

#### 2.1.1. Deacidification of Cranberry Juice

EDBM was performed using a EUR−2C cell (Eurodia, Pertuis, France) with a total effective surface area of 0.14 m^2^. The EDBM configuration used has been described by Faucher et al. [23]. Deacidified CJ samples were collected at DRs of 0% (raw), 19%, 42%, 60%, and 79%, as calculated from titratable acidity measurements, and were analyzed for their physicochemical composition (see below). A sample of raw CJ diluted in distilled water (dilution factor of 4) was included in the study given that consumers are advised to dilute non-deacidified CJ before consumption to avoid gastrointestinal discomfort and other adverse effects. All the CJ samples were filter-sterilized prior to use.

#### 2.1.2. Analysis

##### Titratable Acidity

The titratable acidity of the raw and deacidified CJ samples was measured as described in AOAC method 942.15 [32]. Briefly, the CJ samples were diluted with degassed distilled water, and were then titrated with 0.1 M NaOH until a pH of 8.2 was reached. Titratable acidity is expressed as g/L of citric acid monohydrate equivalents.

##### Organic Acid Content

Organic acids were extracted from the CJ samples using C18-SPE cartridges (non-endcapped, 6 mL, 500 mg; Silicycle, Quebec City, QC, Canada). The cartridges were first conditioned with 5 mL of methanol and were then rinsed with 5 mL of distilled water followed by 10 mL of a 1:1 acetonitrile:water solution. The cartridges were vacuum-dried, and 10 mL of each CJ sample was passed through the cartridges.

The concentrations of quinic acid (QA), citric acid (CA), and malic acid (MA) were determined by HPLC analysis as described in AOAC method 986.13 [33]. Samples (10 µL) were injected on a Synergi Hydro-RP80A column (250 × 4.6 mm; Phenomenex, Torrance, CA, USA) at room temperature using a KH_2_PO_4_ solution (0.2 M, pH 2.4) as an isocratic mobile phase. An Agilent 1100 series HPLC system equipped with a UV detector (wavelength set at 214 nm) was used to separate and detect the organic acids. Calibration curves and the retention times of authentic standards of QA, CA, and MA (Sigma Aldrich, Saint-Louis, MO, USA) were used to quantify and identify the organic acids.

##### Anthocyanin Content

The anthocyanin content of the raw and deacidified CJ samples was measured as described by Wu and Prior [34]. The CJ samples were filtered through 0.45 μm nylon filters, and 20 µL volumes were injected on a Zorbax SB-C18 5 μm column (250 × 4.6 mm, Agilent, Santa Clara, CA, USA) at room temperature. Anthocyanins were eluted with 1 mL/min of two solvents (solvent A: 95% water/5% formic acid and solvent B: 100% methanol). An Agilent 1100 series system equipped with a diode array detector (wavelength set at 520 nm) was used to quantify the anthocyanin content. Results are expressed in mg/L of cyanidin-3-glucoside equivalents.

##### Proanthocyanidin Content

Prior to the determination of the PAC profile, raw and deacidified CJ samples were filtered through 0.45-µm nylon filters. The PACs were quantified as described by Khanal et al. [35]. An Agilent 1260 series HPLC system equipped with a fluorescence detector (emission wavelength: 321 nm, excitation wavelength: 230 nm) was used. Samples (5 µL) were injected on a Develosil 100 Diol-5 column (250 × 4.6 mm; Nomura Chemical Co., Ltd., Kasugai, Japan) at 35 °C. The PACs were eluted based on their degree of polymerization using 0.8 mL/min of two solvents (Solvent A: 98% acetonitrile/2% acetic acid; Solvent B: 95% methanol/3% water/2% acetic acid). An epicatechin calibration curve was used to quantify the PACs using a correction factor to convert the different response factors of monomeric to polymeric PACs. Results are expressed as mg/L of epicatechin equivalents.

##### Total Phenolic Compounds

The concentration of total phenolic compounds was measured using the microscale Folin–Ciocalteu assay [36]. Absorbance was measured using an xMark Microplate spectrophotometer (Bio-Rad Laboratories Inc., Mississauga, ON, Canada) at 765 nm. Results are expressed as mg/L of gallic acid equivalents.

##### Sugar Content

The sugar content of the raw and deacidified CJ samples was determined using the Somogyi–Nelson method at the microplate scale [37]. Absorbance was measured with an xMark Microplate spectrophotometer at 490 nm. Results are expressed as g/L of glucose equivalents.

### 2.2. Bacteria and Growth Conditions

Two cariogenic bacterial species (*Streptococcus mutans* ATCC 25175 and *Streptococcus sobrinus* ATCC 33478) and three commensal bacterial species (*Streptococcus gordonii* ATCC 12559, *Streptococcus oralis* ATCC 35037, and *Streptococcus salivarius* K12) were included in the present study. All streptococcal species were grown aerobically at 37 °C in Brain Heart Infusion broth (BHI; BBL Microbiology Systems, Cockeysville, MD, USA) supplemented with 0.5% (*w/v*) glucose.

### 2.3. Bactericidal Activity against Planktonic Streptococci

The ability of raw and deacidified CJs to kill planktonic streptococci on direct contact was investigated using the NF EN 1040 protocol for disinfectants and antiseptics [38]. First, 10 mL of a 24h culture was vortexed with 200 mg of sterile glass beads (0.3–0.5 mm) to break bacterial chains. The culture was diluted in fresh BHI supplemented with 0.5% glucose to an optical density at 660 nm (OD_660_) of 0.2. Then, 100 µL of the bacterial suspension was added to 900 µL of each of the CJ samples at room temperature. The bacteria were exposed to the CJ samples for 0 min (initial count), 1 min, and 15 min, following which 100 µL of the inoculated CJ samples were added to 900 µL of dilution buffer (0.85% NaCl, 0.01% tryptone; pH 7.0). Ten-fold serial dilutions (10^−3^ to 10^−7^) were prepared, and 100 µL of each dilution was plated on triplicate BHI agar plates supplemented with 0.5% glucose. Dilution buffer was used as a negative control, and chlorhexidine (CHX; 0.12%) was used as a positive control. The plates were incubated for 72 h at 37 °C and colony-forming units (CFU) were determined. Only plates with between 25 and 250 CFU were considered. The experiment was repeated twice.

### 2.4. Bactericidal Activity against Biofilm-Embedded Streptococci

The effect of removing organic acids from CJ on its bactericidal activity against biofilm-embedded streptococci was assessed using FilmTracer LIVE/DEAD Biofilm Viability kit (Life Technologies Corporation, Eugene, OR, USA) according to the manufacturer’s protocol, with slight modifications. The optical density of a 24h bacterial culture was adjusted to OD_660_ = 0.1, and 100 µL were added to the wells of a 96-well clear bottom black wall microplate (Greiner Bio-One North America, Monroe, NC, USA). The microplate was incubated 24 h at 37 °C to allow for biofilm formation. Spent medium and unattached bacteria were removed by aspiration, and the preformed biofilms were exposed to 100 µL of the CJ samples for 1 min and 15 min. The CJ was removed by aspiration, and the biofilms were washed once with 100 µL of Hanks’ Balanced Salt Solution (HBSS; pH 7.2). The biofilms were then treated with 50 µL of a fresh solution of 3 µL/mL of SYTO 9 green (3.34 mM) and 3 µL/mL of propidium iodide (PI; 20 mM) in HBSS for 30 min at room temperature in the dark. The biofilms were rinsed twice with distilled water. Distilled water (100 µL) was added to each well, and relative fluorescence units (Ex: 485 nm/Em: 528 nm for SYTO 9 green, and Ex: 485 nm/Em: 590 nm for PI) were recorded using a Synergy 2 microplate reader (BioTek Instruments, Winooski, VT, USA). Phosphate-buffered saline (PBS; pH 7.2) and 0.12% CHX were used as negative and positive controls, respectively.

### 2.5. Bacterial Adherence to Hydroxyapatite

To assess the adherence of streptococci to saliva-coated hydroxyapatite (HA) preconditioned with CJs, the bottoms of the wells of a 96-well black bottom black wall microplate (Greiner Bio-One North America) were coated with HA according to the protocol described by Shahzad et al. [39]. Briefly, 60 µL of an HA suspension (10% *w/v*, in acetone) was added to each well, and the microplate was incubated at 37 °C for 15 min under constant agitation. The wells were then washed twice with distilled water and once with PBS and were left to dry overnight at 37 °C. Artificial saliva was prepared as described by Madhwani and McBain [40], and 100 µL was added to the HA-coated wells. After 30 min, excess saliva was removed by aspiration, and the HA was preconditioned by adding 100 µL of CJ sample to each well.

Bacterial cells from a 24h culture were labelled with fluorescein isothiocyanate (FITC) as described by Ben Lagha et al. [41]. FITC-labelled bacteria were suspended in PBS to obtain an OD_660_ of 1. Following a 5 min pretreatment of HA-coated wells with the CJ samples, 100 µL of bacterial culture was added to the wells, and the microplate was incubated at 37 °C for 1 h in the dark. The bacteria were removed by aspiration. The wells were washed twice with PBS, and 100 µL of distilled water was added to each well. Fluorescence units of each wells were measured using a Synergy 2 microplate reader with the excitation wavelength set at 485 nm and emission wavelength set at 528 nm. Relative fluorescence units (%) for each treatment were calculated based on the values obtained for the negative control (PBS). Wells with no bacteria were included to determine basal autofluorescence.

### 2.6. Transepithelial Electrical Resistance of Oral Epithelial Barrier

To determine whether exposure to raw and deacidified CJs disrupts the oral epithelial barrier, the immortalized gingival keratinocyte cell line B11, which was previously characterized by Groeger, Michel and Meyle [42], was used to determine tight junction integrity by monitoring transepithelial electrical resistance (TER). The B11 cells were grown at 37 °C in a 5% CO_2_ atmosphere in keratinocyte-serum free medium (K-SFM) supplemented with 50 μg/mL of bovine pituitary extract, 5 ng/mL of human epidermal growth factor, 100 μg/mL of penicillin G-streptomycin, and 2.5 μg/mL of amphotericin B. The B11 cells were seeded at 3.5 × 10^5^ cells/insert in Costar Transwell clear polyester membrane inserts (6.5-mm diameter; 0.4 μm pore size; Corning Co., Cambridge, MA, USA). Supplemented K-SFM was added to the apical (100 µL) and basolateral (600 µL) chambers, and the plate was incubated for 48 h at 37 °C in a 5% CO_2_ atmosphere. The growth medium was then replaced with the same volume of antibiotic-free K-SFM, and the plate was incubated for a further 24 h at 37 °C in a 5% CO_2_ atmosphere to ensure the complete formation of tight junctions. The apical compartment was then filled with 100 µL of raw or deacidified CJ. After 5 min the CJ was removed by aspiration, and 100 µL of antibiotic-free K-SFM was added. The plate was incubated at 37 °C in a 5% CO_2_ atmosphere for the duration of the experiment. TER values were measured using an ohm/voltmeter (EVOM2; World Precision Instruments, Sarasota, FL, USA) at time 0 (initial resistance; before treatment with CJs) and after 1 h, 2 h, 6 h, and 24 h. The results were converted into Ohms (Ω)/cm^2^ by multiplying the resistance with the membrane surface area. A 100% value was attributed to the initial resistance (prior to CJ exposure). The TER measurements are expressed as percentages of the initial TER. The measurements were done in triplicate for each treatment, and the assay was repeated in triplicate. Representative results from one assay are presented.

### 2.7. Fluorescein Isothiocyanate-Conjugated Dextran (FD-4) Transport

The integrity of the oral epithelial barrier was also assessed by measuring the paracellular transport of FITC-conjugated 4.4 kDa dextran (FD-4; Sigma-Aldrich Canada Ltd.) according to a previously described protocol [41]. Briefly, B11 cells were seeded at 3.5 × 10^5^ cells/insert on Costar Transwell clear polyester membrane inserts (6.5-mm diameter; 0.4 μm pore size; Corning Co.) and were treated with CJs as described above. FD-4 (100 µL, 1 mg/mL in K-SFM) and 1.2 mL of K-SFM were added to the apical and basolateral compartments, respectively. Fluorescence in the basolateral chamber was monitored at time 0 and after 1 h, 2 h, 8 h, and 24 h using a Synergy 2 microplate reader with the excitation and emission wavelengths set at 485 nm and 528 nm, respectively. The experiment was repeated in triplicate. Representative results from one experiment are presented.

### 2.8. Immunofluorescent Staining of Zonula Occludens−1 and Occludin

Keratinocytes exposed to raw or deacidified CJ for 5 min were immunostained for zonula occludens−1 (ZO-1) and occludin, two tight junction proteins. B11 keratinocytes were seeded at 3.5 × 10^5^ cells/insert on Costar Transwell clear polyester membrane inserts (6.5-mm diameter; 0.4 μm pore size; Corning Co.) and were treated with CJ samples as described in Section 2.6. Following a 4 h exposure to CJs, the cells were fixed with 100 µL of paraformaldehyde (4% in PBS; Thermo Fisher Scientific, Waltham, MA, USA) at 4 °C for 10 min. The cells were permeabilized by incubating them with 0.1% Triton X−100 (100 µL) for 8 min. After three washes with PBS, 200 µL of freshly prepared blocking buffer (3% skim milk powder, 20 mM TrishCl [pH 8], 150 mM NaCl, and 0.5% Tween 20) was added to the apical chamber and left for 30 min, at 4 °C. Tight junction proteins were stained with 100 µL of either 13 µg/mL of occludin antibody-Alexa Fluor 488 conjugate or 6.5 µg/mL of ZO-1 antibody-Alexa Fluor 594 conjugate (Invitrogen, Waltham, MA, USA) diluted in blocking buffer. The cells were incubated overnight at 4 °C in the dark and were then treated with ProLong Diamond antifade (Life Technologies). An Olympus FSX100 fluorescence microscope and FSX-BSW imaging software (Olympus, Tokyo, Japan) were used to visualize stained ZO-1 and occludin.

### 2.9. Production of IL-6 and IL-8 by Oral Epithelial Cells

To determine whether the removal of organic acids from CJ affected the inflammatory response, GMSM-K oral epithelial cells, which were previously characterized by Gilchrist et al. [43], were seeded at 1.0 × 10^6^ cells/mL in 12-well tissue-culture treated microplates (Sarstedt Inc., Montreal, QC, Canada). The GMSM-K epithelial cell line was used for this assay as it is a producer of pro-inflammatory cytokines, unlike B11 epithelial cells. Following an overnight incubation at 37 °C in a 5% CO_2_ atmosphere, the growth medium was removed by aspiration, and the cells were exposed to 1 mL of raw CJ or deacidified CJ for 5 min at room temperature. A preliminary MTT assay showed that this contact time did not significantly decrease cell viability. The CJ samples were removed by aspiration, and 1 mL of DMEM supplemented with 1% FBS was added to the wells. The cells were incubated at 37 °C for 24 h in a 5% CO_2_ atmosphere. The cell-free culture supernatants were then collected and were kept at –20 °C until used. IL-6 and IL-8 concentrations in the supernatants were determined using enzyme-linked immunosorbent assay (ELISA) kits (eBioscience Inc., San Diego, CA, USA) according to the manufacturer’s protocols.

### 2.10. Statistical Analysis

Unless specified otherwise, all assays were performed in triplicate. Results are expressed as means ± standard deviations (SD). The physicochemical compositions of the CJ samples were analyzed using a one-way analysis of variance (ANOVA) with a posthoc Tukey test (*p* < 0.05). Statistical analyses of bacterial adherence to HA and cytokine secretion were performed using a one-way analysis of variance with a posthoc Bonferroni multiple comparison test (*p* < 0.01) (GraphPad Software Inc., La Jolla, CA, USA). For the other assays, a two-way analysis of variance with a posthoc Bonferroni multiple comparison test (*p* < 0.01) was used.

## 3. Results

### 3.1. Composition of the Raw and Deacidified CJs

EDBM is an electrochemical process that selectively removes CA and MA from CJ, with a reduction in QA content when the DR exceeds 40% [25]. As can be seen in Table 1, there was a linear decrease in CA and MA throughout the deacidification process, leading to the complete elimination of MA in the CJ sample deacidifed at a rate of 79%. The release of OH^−^ ions in the juice compartment by the bipolar membranes during the process concurrently caused the pH of the CJ to increase from 2.6 (raw CJ) to 3.2 (DR 79%). However, as expected based on the literature [23,25], the concentrations of sugar, total phenolic compounds, anthocyanins, and PACs remained unchanged in all the raw and deacidified CJ samples. The pH of the 1/4 dilution of raw CJ was 2.6 (the same as undiluted raw CJ), and it was expected that the diluted raw CJ contained 25% of the sugar, total phenolic, and organic acid content of raw CJ.

### 3.2. Bactericidal Activity of the Raw and Deacidified CJs against Planktonic Bacteria

**Figure 1:**
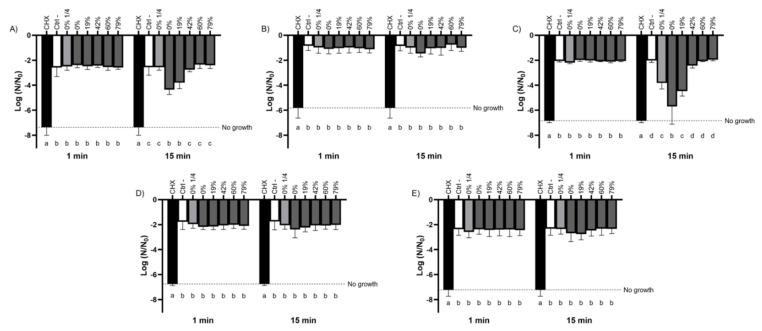
Effect of the deacidification rate (0, 19, 42, 60, and 79%) of cranberry juice on the viability of planktonic oral streptococci reported as log of the initial bacterial count (N_0_) following 1-min and 15-min contacts. The effect of a ¼ dilution of raw CJ on bactericidal activity was also studied. (**A**) *S. mutans*, (**B**) *S. sobrinus*, (**C**) *S. gordonii*, (**D**) *S. oralis*, and (**E**) *S. salivarius*. Results are expressed as mean log (N/N_0_) ± SD of triplicate assays from two independent experiments. Columns with different letters are significantly different from one another (Two-way ANOVA, Bonferroni test, *p* < 0.01). CHX: Chlorhexidine 0.12%. Ctrl: Dilution buffer.

The bactericidal activity of the raw CJ and deacidified CJs was evaluated using two cariogenic and three commensal species of oral streptococci. As shown in Figure 1, a short contact time (1 min) with the CJ samples did not result in an increase in bactericidal activity for any of the streptococci studied compared to the negative control. However, the susceptibility of the bacteria to the CJ samples differed between species when the exposure time was extended to 15 min. While the bactericidal activity of the CJ samples against *S. sobrinus* (Figure 1B), *S. oralis* (Figure 1D), and *S. salivarius* (Figure 1E) were not significantly different from that of the negative control, *S. mutans* (Figure 1A) and *S. gordonii* (Figure 1C) populations decreased with the DR of the CJ samples. In the case of *S. mutans*, undiluted raw CJ caused a mean log reduction of 4.36, whereas a 19% DR CJ caused a 3.80 log reduction in the viable bacterial population. Neither DRs ≥42% nor a 1/4 dilution of raw CJ exhibited significantly different bactericidal properties compared to the dilution buffer, which suggests that the observed effects are linked to the concentration of organic acids more than to the low pH of the samples. Similar results were obtained for *S. gordonii*, which had a bacterial mortality close to that of the control after a 15 min exposure for DRs ≥42%. However, a 1/4 dilution of raw CJ reduced the viability of *S. gordonii* to the same extent as a 19% DR CJ sample, suggesting the impact of low pH on the bactericidal activity of the CJ against this species. CHX, which was used as a positive bactericidal agent, quickly eradicated all the bacteria studied for the two test times (1 min and 15 min).

### 3.3. Bactericidal Activity of the Raw and Deacidified CJs against Biofilm-Embedded Bacteria

**Figure 2:**
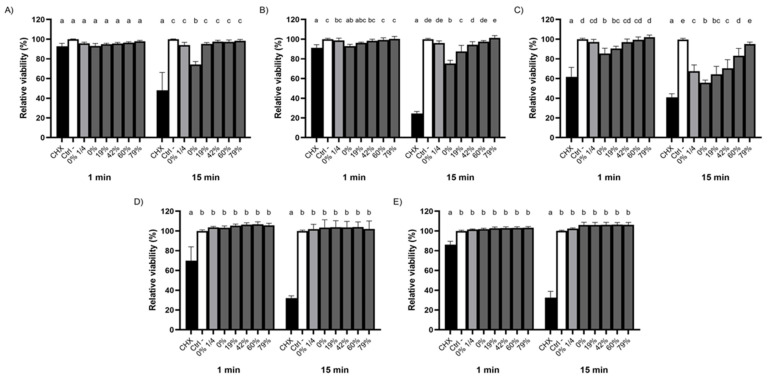
Effect of the deacidification rate (0, 19, 42, 60, and 79%) of cranberry juice on the killing of biofilm-embedded (**A**) *S. mutans*, (**B**) *S. sobrinus*, (**C**) *S. gordonii*, (**D**) *S. oralis*, and (**E**) *S. salivarius* following 1- and 15-min exposures. A ¼ dilution of raw CJ was also included. Cell viability was assessed using FilmTracer LIVE/DEAD Biofilm Viability kit. A 100% value was attributed to the negative control (PBS). Results are expressed as means ± SD of triplicate assays from three independent experiments. Columns with different letters are significantly different from one another (Two-way ANOVA, Bonferroni test, *p* < 0.01). CHX: Chlorhexidine 0.12%. Ctrl: PBS.

The extent to which the removal of organic acids from CJ impacts its bactericidal activity was further investigated using oral streptococci embedded in a biofilm (Figure 2). It required 15 min for undiluted raw CJ to significantly affect the viability of a *S. mutans* biofilm, lowering its viability rate to 74.3 ± 3.0%. On the other hand, DRs ≥19% suppressed this effect as did a 1/4 dilution of raw CJ (Figure 2A). Although significant differences were obtained between DRs for biofilm-embedded *S. sobrinus* after 1 min, viability rates were above 91% for all the CJ samples and even CHX, which indicated that biofilm-embedded *S. sobrinus* is resistant over the very short term (Figure 2B). However, the sensitivity of *S. sobrinus* biofilms to CJ increased with an extended exposure time, with viability rates dropping to 75.2 ± 3.4% for undiluted raw CJ and 87.5 ± 6.2% for the 19% DR CJ. The viability rates remained similar to that of PBS for DRs ≥42% after a contact time of 15 min. The bactericidal activity of raw and 19% DR CJs against biofilm-embedded *S. gordonii* manifested quickly, as shown by the significant loss in relative viability after 1 min (Figure 2C). However, the linear relationship between higher DRs and the viability rates of biofilm-embedded *S. gordonii* was more marked after a 15 min exposure. The viability rates for different DRs fell to 55.7 ± 2.7% (undiluted raw CJ), 64.3 ± 8.0% (19% CJ), 70.3 ± 8.8% (42% CJ), 83.2 ± 7.5% (60% CJ), and 95.0 ± 2.0% (79% CJ), the effect of 79% CJ being statistically similar to the negative control. In addition, the calculated viability rate for the 1/4 dilution of raw CJ (67.4 ± 6.4%) was comparable to that of 19% CJ. Lastly, as was observed with planktonic bacteria, the viability of biofilm-embedded *S. oralis* and *S. salivarius* was not affected regardless of the DR, although CHX did significantly decrease their viability (Figure 2D,E). Overall, our results suggest that the raw and deacidified CJ samples exhibited antibacterial activities that were species-specific. In all cases, however, the viability of all the biofilm-embedded streptococcal species tested was more affected by CHX than by the CJ samples.

### 3.4. Impact of CJ Deacidification on Bacterial Adherence to Saliva-Coated HA

**Figure 3:**
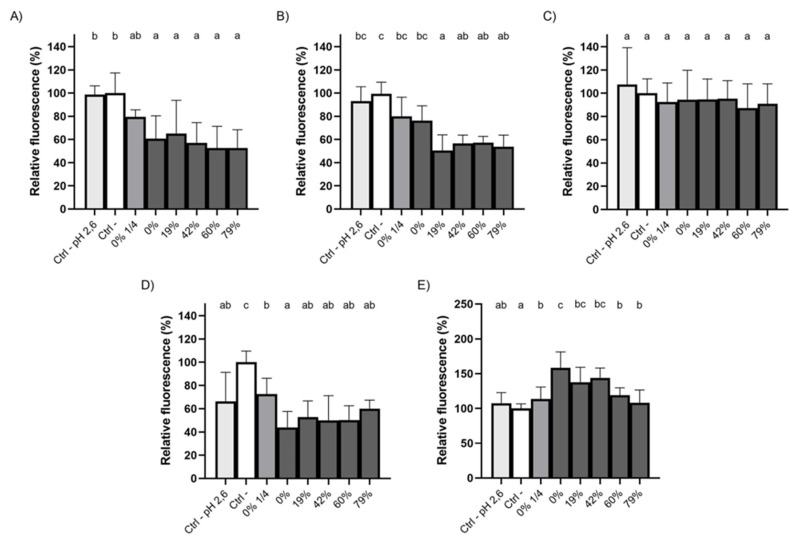
Adherence of FITC-labelled (**A**) *S. mutans*, (**B**) *S. sobrinus*, (**C**) *S. gordonii*, (**D**) *S. oralis*, and (**E**) *S. salivarius* to saliva-coated HA preconditioned (5 min) with raw or deacidified (0, 19, 42, 60, and 79%) cranberry juice. A 100% value was attributed to the negative control. Results are expressed as means ± SD of triplicate assays from three independent experiments. Columns with different letters are significantly different from one another (ANOVA, Bonferroni test, *p* < 0.01). Ctrl: PBS.

Given that the formation of dental plaque and the process of dental caries rely on bacterial colonization of oral surfaces, we investigated the ability of raw and deacidified CJ samples to hinder bacterial adherence to HA, the primary mineral of dental enamel. Pre-conditioning saliva-coated HA with undiluted CJ samples hindered the adherence of *S. mutans*, regardless of the DR (Figure 3A), with the average fluorescence units measured being 1.5- to 1.9-times lower than the negative control (PBS, pH 7.2) following a 1h incubation. No significant differences were observed between CJ samples. The 1/4 dilution of raw CJ led to a relative adherence of 79.4 ± 6.1% compared to 60.6 ± 20% for undiluted raw CJ. Overall, this shows that the phenolic compounds in the CJ samples, whose concentration remained the same in the undiluted CJ samples throughout the EDBM process (Table 1), were likely responsible for the decrease in adherence observed for *S. mutans*. A comparison with the results obtained for PBS at pH 2.6, which were not significantly different from the control (PBS at pH 7.2), supports this hypothesis. Similar results were obtained for *S. sobrinus* (Figure 3B) and *S. oralis* (Figure 3D), although, for *S. oralis*, the low pH of the CJ samples may have contributed, in part, to the reduction in bacterial adherence given that a significant decrease was observed for PBS at pH 2.6 (33% lower fluorescence) compared to PBS at pH 7.2. *S. gordonii* adhered to HA surfaces to the same extent for all the CJ samples and controls (Figure 3C), which suggests that the cranberry phenolic compounds did not impair the adherence of this bacterial species. Interestingly, unlike the other streptococci tested, the adherence of *S. salivarius* to saliva-coated HA significantly increased following the conditioning of the HA surfaces with undiluted raw, 19% DR or 42% DR CJs (Figure 3E). Indeed, the measured fluorescence for these three CJ samples was 1.4- to 1.6-times higher than for PBS (pH 7.2). The low pH of the CJ samples did not appear to play a role in adherence to HA surfaces based on the relative fluorescence obtained with PBS at pH 2.6.

### 3.5. Effect of CJ Deacidification on Oral Epithelial Barrier Integrity

**Figure 4:**
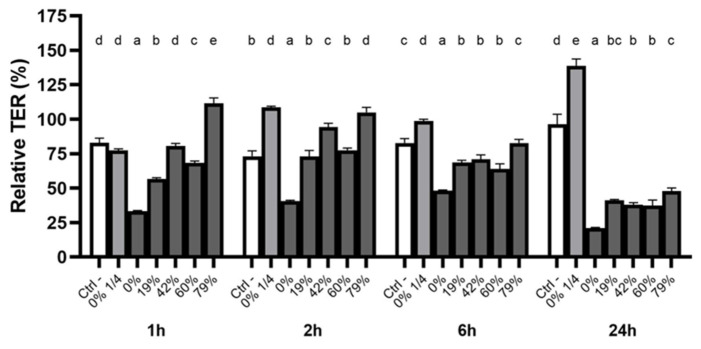
Time-dependent relative TER measurements of a B11 keratinocyte monolayer exposed (5 min) to raw or deacidified (0, 19, 42, 60, and 79%) cranberry juice. The basal TER of each well was measured before the exposure to cranberry juice (t_0_). The results are expressed as means ± SD of % of TER at t_0_ (*n* = 3 from a representative experiment). Columns with different letters are significantly different from one another (Two-way ANOVA, Bonferroni test, *p* < 0.01). Ctrl: antibiotic-free K-SFM.

The oral epithelial mucosa is directly exposed to food and drink. It is thus of particular importance to determine whether the deacidification of CJ can help preserve the integrity of the oral epithelial barrier. A correlation between higher DRs and the maintenance of TER was observed shortly after a 5-min exposure to undiluted CJs (1–2 h follow-up) (Figure 4). Undiluted raw CJ reduced the relative TER to 33.4 ± 0.4% 1h post-exposure, whereas TER dropped to 56.7 ± 0.8% with a 19% DR CJ. A DR 79% CJ had a protective effect over the short term, with the relative TER reaching 111.4 ± 4.0% 1h post-exposure and 104.5 ± 3.6% 2h post-exposure. These values were significantly higher than the control values in both cases. After a 6h exposure to the CJ samples, the relative TER following exposure to raw CJ remained below that of all the deacidified CJ samples. The TER following exposure to the 79% DR CJ was higher than that of the other undiluted samples. No significant differences were observed for the 19%, 42%, and 60% DR CJ samples. Nonetheless, the tight junction integrity of oral keratinocytes treated with the undiluted CJ samples did not recover after 24 h, with the relative TER ranging from 21 to 47%. However, the barrier function strengthened within 2 h following exposure to a 1/4 dilution of raw CJ, and the TER reached 138.6 ± 5.2% of its initial value after 24 h. The barrier integrity of the keratinocytes treated with growth medium (control) was restored after 24 h (relative TER of 96.3 ± 7.2%).

### 3.6. Impact of CJ Deacidification on the Paracellular Transport of FD-4

**Figure 5:**
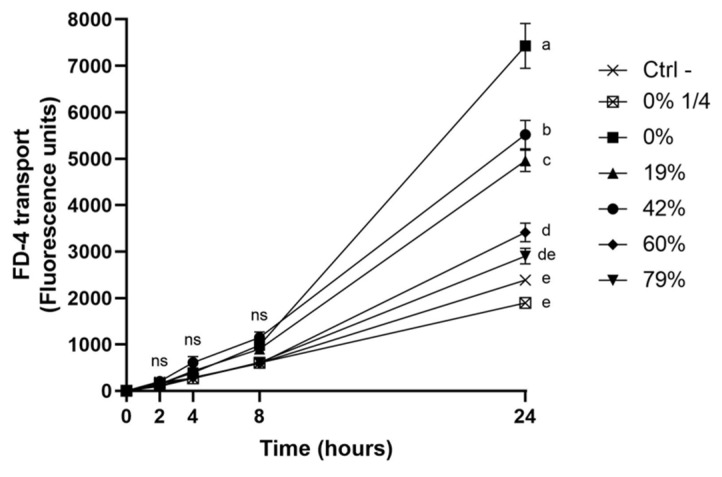
Time-dependent paracellular transport of FD-4 across a B11 keratinocyte monolayer exposed (5 min) to raw or deacidified (0, 19, 42, 60, and 79%) cranberry juice. FD-4 was added to the apical compartment, and the fluorescence of the basolateral compartment is reported. Results are expressed as means ± SD of triplicate assays from a representative experiment. Treatments with different letters are significantly different from one another (Two-way ANOVA, Bonferroni test, *p* < 0.01).

The modulation of the integrity of the oral epithelial barrier by the deacidification of CJ was further investigated by monitoring the paracellular transport of FD-4 after a 5-min exposure to the CJ samples (Figure 5). The reduction in the permeability of the keratinocyte monolayer caused by deacidified CJ samples was correlated with the increase in DR. However, significant differences among CJ samples were only noted 24h post-exposure. Although the permeability of the keratinocyte barrier exposed to the 79% DR CJ did not significantly differ from the control treatment, all other DRs caused an increase in permeability. Undiluted raw CJ led to an average FD-4 transport that was 3.1-times higher than that of the control, while a 1/4 dilution of raw CJ had an effect on barrier integrity similar to that of the control. The results of FD-4 paracellular transport, together with the results of the TER measurements, suggest that organic acids could weaken the oral epithelial barrier and that this deleterious effect was lessened by CJ deacidification.

### 3.7. Immunofluorescence Staining of ZO-1 and Occludin Following Exposure to CJ Samples

**Figure 6:**
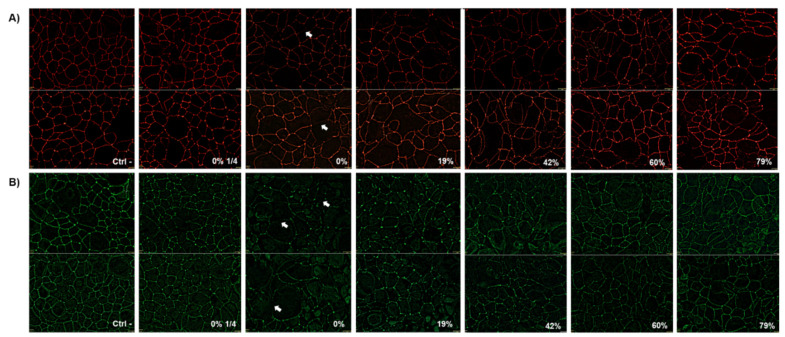
Immunofluorescence staining of (**A**) zonula occludens−1 (ZO-1) and (**B**) occludin in B11 oral keratinocytes 24 h after being challenged by raw or deacidified (0, 19, 42, 60, and 79%) cranberry juice for 5 min. Images from two independent experiments are presented.

To determine whether the disruption of epithelial barrier integrity was due to a modification in tight junction protein distribution, ZO-1 and occludin were immunostained 24 h after exposure to raw and deacidified CJ samples. As shown in Figure 6A, a brief 5-min contact with undiluted raw CJ had a mild effect on ZO-1 labeling. Slight discontinuities in paracellular ZO-1 were observed with this sample, as indicated by the arrows. Moreover, cell morphology was altered following the contact with the undiluted CJ samples, and treated keratinocytes appeared enlarged compared to the control cells. However, a more apparent impact on occludin distribution was observed with keratinocytes treated with undiluted raw CJ (Figure 6B). Discontinuities in the labeling of occludin and visible change in cell morphology were observed following exposure to undiluted raw CJ sample in comparison to the control.

### 3.8. Effect of CJ Deacidification on IL-6 and IL-8 Production by Oral Epithelial Cells

**Figure 7:**
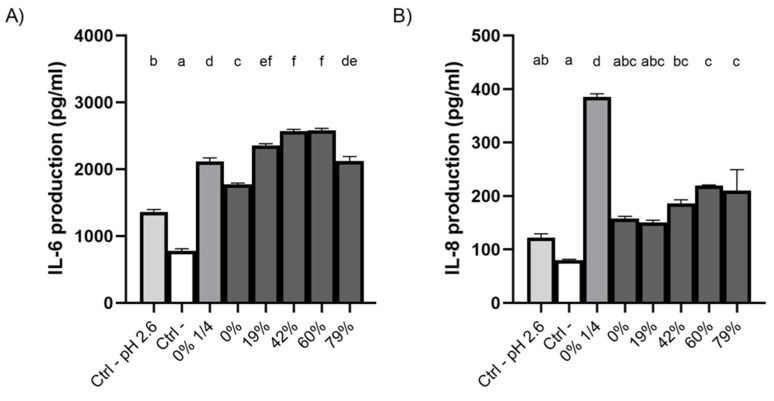
Effect on the production of (**A**) IL-6 and (**B**) IL-8 of a 5-min exposure of GMSM-K oral epithelial cells to raw or deacidified (0, 19, 42, 60, and 79%) cranberry juice. A ¼ dilution of raw CJ was also included. Cells were incubated for 24 h post-exposure, following which the supernatants were collected and the secretion of cytokines was measured. Results are expressed as means ± SD of a triplicate assay. Columns with different letters are significantly different from one another (ANOVA, Bonferroni test, *p* < 0.01). Ctrl: DMEM+1% FBS.

The production of the pro-inflammatory cytokines IL-6 and IL-8 by oral epithelial cells treated with CJ samples was investigated using the GMSM-K cell line (Figure 7). All the raw and deacidified CJ samples caused an increase in the production of IL-6 by epithelial cells compared to the negative control (DMEM + 1% FBS) (Figure 7A), which could be partly explained by the low pH of the samples. Indeed, epithelial cells subjected to a sample of growth medium whose pH has been adjusted to 2.6 with HCl produced more IL-6 than the negative control (neutral pH). Additionally, CJ samples with DRs ≥19% were more inflammatory than undiluted and diluted raw CJ samples. However, there was a significant decrease in IL-6 secretion when the DR reached 79%, compared to 60% DR. The production of IL-8 by oral epithelial cells treated with the CJ samples was not significantly affected by the DR, although an increase in production was observed at higher DRs (Figure 7B). Moreover, the raw and 19% DR CJ samples had no effect on the production of this chemokine compared to the negative control. The low pH of CJ was not a triggering factor in the secretion of IL-8 by oral epithelial cells as there was no significant difference in IL-8 concentrations between the neutral pH growth medium and the pH-adjusted growth medium (pH 2.6). 

## 4. Discussion

### 4.1. Bactericidal Activity of Raw and Deacidified CJs against Planktonic and Biofilm-Embedded Streptococci

A study by Svensäter et al. [44] showed that a pH between 3.0 and 3.5 kills the streptococci tested in the present study. However, the raw and deacidified CJ samples, with pHs ranging from 2.6 to 3.2 (Table 1), did not cause the complete eradication of the planktonic bacteria. The high sugar content of the undiluted CJ samples at all DRs (average of 46.9 ± 6.5 g/L of glucose equivalents) may have provided some protection to the bacteria. Indeed, Sheng and Marquis [45] reported that a 5 g/L concentration of glucose can attenuate the death of oral streptococci under acidic stress (at pH 3.0 for *S. mutans* and *S. sobrinus*, and pH 3.5 for *S. gordonii*). A second factor that may have protected the bacteria from the acidic effect of the CJ samples is the ability of mutans streptococci to mount an acid tolerance response (ATR), which allows them to maintain an active metabolism under sub-lethal pH conditions [46,47]. Mutans streptococci can mount an ATR when they are grown in glucose-enriched medium, which promotes the production of glucans [48]. The BHI broth medium was supplemented with 0.5% glucose, which would thus have further increased the aciduricity of the bacteria.

The present study revealed that there is a significant reduction in planktonic bacterial populations of *S. mutans* exposed for 15 min to undiluted raw and 19% DR CJs. Although *S. mutans* is an acid-tolerant bacteria, citrate (1.5 g/L) has been reported to enhance the killing of planktonic *S. mutans*, especially at low pH values [49,50]. Furthermore, the ATR of *S. mutans* is characterized by an increase in F_1_F_0_-ATPase activity, which helps maintain an internal pH 0.5 to 1.0 unit more alkaline than the external pH [46,47,51]. However, Duarte et al. [18] showed that a cranberry extract (20 mg/mL) containing 300–500 µg/mL of PACs inhibits the ATPase activity of *S. mutans* by 80%, whereas Gregoire et al. [19] reported that cranberry flavonoids (500 µM), taken individually and in combination, cause an 18–33% reduction in F-ATPase activity. Given this, the phenolic content of CJ may have impaired the protective mechanisms inherent to *S. mutans*, which may have led to the fast decrease in bacterial populations at low DRs.

In the case of *S. sobrinus*, planktonic bacterial cells survived 1-min and 15-min exposures to all the CJ samples, which contradicts the work of Kranz et al. [52], who reported that a 1-min immersion in undiluted CJ completely eliminates this bacterium. This discrepancy may be explained by the different growth media used. Nascimento et al. [53] suggested that *S. sobrinus* can mount an ATR by increasing the activity of the glucose phosphoenolpyruvate:sugar phosphotransferase system (PTS) when grown in a pH 5 environment. The Tryptone Soy Broth used by Kranz et al. [52] was not enriched in glucose, unlike the BHI used in the present study, which was supplemented with 0.5% glucose. The lack of glucose enrichment may have prevented the production of large amounts of acids by glycolysis during the growth of the bacterium, and may thus have prevented *S. sobrinus* from mounting an ATR that could have protected it during the exposure to CJ. *S. sobrinus* also relies on an increase in malolactic fermentation to protect itself in low pH environments [53,54]. It should be noted here that the CJ samples contained L-malate when the DR was ≤60%. It has also been reported that the F-ATPase activity of *S. sobrinus* remains stable or increases slightly under acidic stress [53,54], meaning that phenolic compounds in CJ do not hinder its ability to survive the 15-min exposure, regardless of the DR. No bactericidal activity of CJ diluted to 1/4 against planktonic *S. mutans* and *S. sobrinus* has been reported in the literature [20,52].

Svensäter et al. [44] reported that *S. gordonii* is the least acid-tolerant streptococcus, which corroborates the results obtained with the streptococci tested in the present study. The survival of planktonic *S. oralis* and *S. salivarius* that we observed suggests that they could survive in an acidic environment, at least over the short term. This could be linked to the activity of the arginine deiminase system (ADS), which increases the resistance of the bacteria at pH 3.5 [55,56,57].

All the streptococci tested possess one or more genes encoding GTFs [11], which enables them to produce an EPS matrix using the glucose in the growth medium [58]. The biofilm-embedment of streptococci in an EPS matrix is crucial as it slows down the diffusion of charged or high molecular weight compounds such as protons (H^+^) and anionic forms of organic acids, and creates stable microenvironments in the form of microcolonies [12]. Hwang et al. [12] subjected a preformed *S. mutans* biofilm to matrix degradation using dextranase and showed that the pH of the *S. mutans* biofilms, which was highly acidic prior to the degradation of the EPS, is neutralized after a 60-min exposure to Na_2_HPO_4-_citric acid buffer (pH 7), whereas the pH increases only in the top layer of the biofilm in the presence of a neutral buffer when the EPS was left intact [12]. It may thus be possible that the decrease in biofilm viability observed for *S. mutans* and *S. sobrinus* following a 15-min exposure to undiluted raw CJ affected the upper layer more than the bottom layer of the biofilms [12,59]. A study by Gilmore et al. [58] showed that an early-formed *S. gordonii* biofilm (<4 days) lacks a complex structure and bacterial clusters. Consequently, the viability rates of 24h *S. gordonii* biofilms exposed to CJ samples, which decreased linearly with the increase in DR, may be the result of a rather loose EPS matrix that does not substantially hinder the diffusion of the organic acids in the CJ [60,61]. On the other hand, EPS-embedded microcolonies have been observed by confocal microscopy in 24h *S. oralis* biofilms [62]. This type of biofilm structure may explain why the viability of *S. oralis* was not affected by the CJ treatments.

### 4.2. Effect of CJ Deacidification on the Adherence of Streptococci to Saliva-Coated HA

Plant polyphenols found in various beverages reduce the adherence of mutans streptococci to enamel either in vitro or in situ upon consumption [20,63]. Koo et al. [20] observed a 6.2-fold decrease in the adherence of *S. mutans* to salivary-coated HA preconditioned with a 1/4 diluted CJ compared to pretreatment with a control devoid of phenolic compounds and containing only CJ sugars and organic acids. Interestingly, a 30-min pretreatment of bacterial suspensions with a CJ whose pH had been adjusted to 5.5 does not hamper the attachment of *S. mutans* to HA beads [20]. This suggests that the phenolic compounds in CJ hinder bacterial adherence through interactions with salivary proteins more than with bacterial cell surface proteins. Weiss et al. [64] reported that non-dialyzable CJ material has a similar effect on the adherence of *S. sobrinus* to HA. The results of the present study indicate that a 1/4 dilution of raw CJ increased the attachment of *S. mutans* and *S. sobrinus* to HA compared to undiluted CJs, further reflecting the role that cranberry polyphenols play in the anti-adherence properties of the beverage. The adherence of mitis streptococci such as *S. gordonii* and *S. oralis* mainly relies on binding to sialic acid and salivary glycoproteins such as mucins [2,65,66]. These early colonizers possess diverse lectins and use various mechanisms to adhere to HA [2,3,4,67,68]. The CJ samples could have affected bacterial colonization of HA surfaces in a species-specific way, which would explain the differences observed with the adherence of commensal streptococci to HA. The increase in the adherence of *S. salivarius* to saliva-coated HA may be more linked to its ability to auto-aggregate than to an enhancement of bacterial adherence driven by CJ [69].

### 4.3. Effect of CJ Deacidification on Oral Epithelial Barrier Function

The maintenance of the integrity of the oral mucosa is key to avoid bacterial invasion of the underlying connective tissue. The results from the present study show that deacidification of CJ protected tight junction integrity in an oral keratinocyte model shortly after contact with the beverage as determined by the analyses of TER and the paracellular transport of FD-4. Serre et al. [30] proposed that a minimum DR of 37% is required to improve the maintenance of intestinal barrier integrity in vitro compared to non-deacidified CJ. The results from the present study suggest that a DR of 19% was sufficient to have a beneficial impact on the oral keratinocyte barrier. However, the batch of raw CJ used by Serre et al. [30] contained higher concentrations of citric acid (1.9 times more) and malic acid (2.3 times more) than the CJ we used in the present study, which may justify the need for a higher DR. Furthermore, Renaud et al. [31], who investigated how the type of organic acid removed from CJ impacts the TER of a Caco-2 intestinal cell monolayer, provided evidence that citrate contributes to the disruption of the epithelial barrier more than malate. Okada et al. [70] showed that the permeability of the vaginal epithelium of rats treated with acidic solutions for 1 h is enhanced by solutions with a lower pH (3.5 instead of 6.6), higher concentrations of organic acid, and the stronger chelating ability of the acid they contain (citrate > malate). This could be attributed to the presence of three binding sites on the molecular structure of citrate, as opposed to two for malate, which means that citrate binds more calcium ions than malate [71]. As Ca^2+^ is essential for localizing tight junction proteins such as ZO-1 and occludin at cell-cell contact sites [72], this means that the citrate in CJ can damage the integrity of the epithelial barrier. The beneficial impact of CJ deacidification on tight junction integrity observed for the first 6 h following exposure probably relies on the selective removal of up to 80% of the citrate from CJ by EDBM.

Okada et al. [70] showed that the integrity of the rat vaginal epithelial barrier recovers 2 h after exposure. This contradicts our results with the oral keratinocyte model showing the inability of oral keratinocytes to recover from the acidic stress caused by the CJ samples even after 24 h. This was likely due to the high sugar content of the undiluted CJ samples (Table 1), which could also explain the change in cell morphology that was observed. Yu et al. [73] treated Caco-2 intestinal epithelial cells at confluence with a high concentration of glucose (135 g/L) for 60 min, and reported that the permeability of the epithelial barrier to FITC-conjugated 70 kDa dextran increases two-fold compared to the control. A decrease in epithelial barrier integrity caused by glucose (3.6 g/L) through a reduction in TER has also been reported by Mongelli-Sabino et al. [74] for the MDCK kidney epithelial cell line. Moreover, the maintenance of the epithelial barrier integrity following the treatment with 1/4 diluted raw CJ at all the times tested, compared to undiluted CJ samples, may have been caused by the lower glucose content resulting from the dilution of the raw CJ with distilled water. It is noteworthy that Yu et al. [73] associated the noticeable swelling of Caco-2 cells they observed with the activation of the Na^+^/glucose cotransporter by a glucose solution (135 g/L). This cotransporter may also be involved in glucose absorption by human oral mucosa ex vivo following a 5-min exposure to glucose [75], indicating that the change in keratinocyte morphology observed by immunostaining may be caused by the glucose content of undiluted CJ. 

However, it should be mentioned that our results were obtained in a static oral epithelial barrier model devoid of salivary glands, which is not representative of the complex physiological response that occurs following gustatory stimulation with food and beverages. Studies comparing static and dynamic in vitro models of the intestinal barrier and the blood–brain barrier showed that the constant flow of growth medium in dynamic models preserved the integrity and reduced the permeability of the barriers [76,77]. Based on these studies, and especially considering that acids contained in food greatly stimulate the salivary flow in the oral cavity [78], it is likely that the reported impact of CJ deacidification on the oral barrier integrity would not be as significant following human consumption of the juice. Further experiments are required to confirm the benefits of CJ deacidification on the oral mucosa in vivo.

### 4.4. Effect of CJ Deacidification on the Production of IL-6 and IL-8 by Oral Epithelial Cells

A 5-min contact with raw and deacidified CJs induced a significantly higher secretion of IL-6 by oral epithelial cells compared to the negative control. Based on reports in the literature, the organic acid composition and sugar content of CJ may modulate the overproduction of this pro-inflammatory cytokine. Held et al. [79] showed that a 6h exposure to orange juice diluted 1:10 induces 22% higher IL-6 production by KB epithelial cells than untreated cells and that approximately half of the increase is due to the organic acid content (as with CJ, orange juice is rich in CA and MA), while the other half is related to the sugar fraction of the orange juice. Interestingly, they reported that there is no increase in the inflammatory response when the KB cells are treated with growth medium with a pH of 4.2. However, we showed that GMSM-K epithelial cells produce more IL-6 when exposed to DMEM at pH 2.6 than to the neutral control. This discrepancy may be caused by differences in cell lines and in pH values. In addition, epithelial cells are hypertonically stressed by the carbohydrates in CJ. For instance, a 2h treatment of Caco-2 intestinal epithelial cells in a hyperosmotic environment causes an overexpression of the gene encoding IL-6 [80].

The deacidification of CJ also had an impact on the production of the pro-inflammatory chemokine IL-8. The significant differences observed between the negative control and all the CJ samples with different DRs may have been caused by the osmolarity of the CJ samples. Grauso et al. [80] used hyperosmotic solutions of mannitol (30 g/L and 60 g/L) to investigate how osmotic pressure impacts the intestinal barrier in vitro. They reported that, compared to an isotonic control, the expression of the *CXCL8* gene in Caco-2 cells increases 14.2-fold and 4.8-fold when the cells are exposed for 2 h to the 30 g/L mannitol solution and the 60 g/L mannitol solution, respectively [80]. This indicates that a lower hypertonic stress induces a higher production of IL-8, which is in accordance with our results with 1/4 diluted CJ and undiluted CJs for GMSM-K cells. The results of Lan et al. [81], who measured a 1.5-times higher concentration of IL-8 in the supernatant of keratinocytes exposed to 26 mM (4.7 g/L) glucose for 7 days are in line with this explanation.

## 5. Conclusions

Overall, the deacidification process had little impact on the bactericidal activities of CJ against the five species of streptococci tested compared to the raw product. Apart from *S. mutans* (planktonic) and *S. gordonii* (planktonic and biofilm-embedded), bacterial viability was only slightly impacted by a 15-min contact with the CJ samples, regardless of the organic acid profile. Furthermore, all the undiluted CJ samples reduced the adherence of the two cariogenic species tested to saliva-coated HA to an equal extent, while they did not impair the adherence of *S. gordonii* and *S. salivarius*. Although the 1/4 dilution of raw CJ lowered the antibacterial properties of CJ against *S. mutans* and *S. sobrinus*, the change was not significant. We used an in vitro oral epithelium model to show that removing organic acids had a beneficial effect as it dampened the disruption of the epithelial barrier function induced by the raw CJ. This appeared to be related to the maintenance of tight junction protein integrity. When the raw CJ was diluted in water prior to consumption, as recommended by the manufacturer, it caused significantly less damage in the in vitro oral epithelium model due to the reduction in its glucose content. However, the dilution of the raw CJ caused a spike in IL-8 secretion by oral epithelial cells. Based on these results, the deacidification of CJ appears to be a promising compromise as it minimized the adherence of cariogenic species and maximized the maintenance of epithelial barrier integrity. Further studies using in vivo models are required to determine whether this conclusion holds true in the human oral cavity.

## Figures and Tables

**Table 1:** Physicochemical composition of the raw and deacidified cranberry juices.

Deacidification Rate (%)	0 (raw)	19	42	60	79
pH	2.59 ± 0.03 ^a^	2.74 ± 0.01 ^b^	2.71 ± 0.01 ^b^	2.87 ± 0.01 ^c^	3.24 ± 0.02 ^d^
Titrable acidity (g/L of citric acid monohydrate equivalents)	9.25 ± 0.05 ^a^	7.48 ± 0.02 ^b^	5.40 ± 0.05 ^c^	3.72 ± 0.02 ^d^	1.91 ± 0.05 ^e^
Sugar (g/L of glucose equivalents)	44.4 ± 4.7 ^a^	44.1 ± 6.6 ^a^	44.9 ± 5.1 ^a^	47.8 ± 10.3 ^a^	53.3 ± 2.5 ^a^
Organic acids (g/L)					
Quinic acid	10.35 ± 0.31 ^a b^	10.72 ± 0.04 ^a^	10.49 ± 0.17 ^a b^	10.11 ± 0.14 ^b^	9.19 ± 0.11 ^c^
Citric acid	11.59 ± 0.20 ^a^	9.49 ± 0.15 ^b^	6.88 ± 0.15 ^c^	4.67 ± 0.09 ^d^	2.35 ± 0.06 ^e^
Malic acid	6.03 ± 0.10 ^a^	4.44 ± 0.04 ^b^	2.40 ± 0.11 ^c^	1.34 ± 0.06 ^d^	0.00 ± 0.00 ^e^
Anthocyanins (mg/L of cyanidin−3-glucoside equivalents)					
Cyanidin−3-galactoside	65.14 ± 0.51 ^a^	65.59 ± 0.67 ^a^	64.70 ± 0.32 ^a^	65.76 ± 1.22 ^a^	61.89 ± 0.61 ^b^
Cyanidin−3-glucoside	2.15 ± 0.12 ^a^	2.97 ± 0.06 ^b^	2.47 ± 0.10 ^c^	2.06 ± 0.06 ^a^	2.22 ± 0.19 ^a c^
Cyanidin−3-arabinoside	51.12 ± 0.69 ^a^	51.27 ± 0.15 ^a^	50.99 ± 0.37 ^a^	50.57 ± 0.89 ^a^	48.24 ± 0.22 ^b^
Peonidin−3-galactoside	84.74 ± 0.54 ^a^	85.91 ± 0.95 ^a^	83.76 ± 0.51 ^a^	85.02 ± 1.21 ^a^	80.74 ± 0.71 ^b^
Peonidin−3-glucoside	8.50 ± 0.10 ^a^	8.86 ± 0.09 ^b c^	9.01 ± 0.12 ^b^	8.71 ± 0.06 ^a b c^	8.53 ± 0.10 ^a c^
Peonidin−3-arabinoside	37.94 ± 0.62 ^a^	38.41 ± 0.39 ^a^	37.13 ± 0.36 ^a^	37.29 ± 0.34 ^a^	35.98 ± 0.30 ^b^
Total	249.58 ± 1.82 ^a^	253.11 ± 0.94 ^a^	248.05 ± 0.51 ^a^	249.40 ± 2.75 ^a^	237.60 ± 1.47 ^b^
Proanthocyanidins (mg/L of epicatechin equivalents)					
Monomers	39.35 ± 0.64 ^a^	40.43 ± 1.35 ^a^	36.05 ± 2.74 ^a^	36.67 ± 2.41 ^a^	37.43 ± 1.97 ^a^
2–3mers	148.36 ± 1.80 ^a^	155.41 ± 6.66 ^a^	142.12 ± 18.47 ^a^	157.39 ± 3.86 ^a^	159.30 ± 9.67 ^a^
4–6mers	59.92 ± 1.24 ^a^	62.94 ± 2.26 ^a^	57.64 ± 7.06 ^a^	62.55 ± 1.88 ^a^	62.29 ± 3.58 ^a^
7–10mers	4.28 ± 0.27 ^a^	4.52 ± 0.35 ^a^	4.06 ± 0.54 ^a^	4.41 ± 0.48 ^a^	4.53 ± 0.50 ^a^
Polymers	5.55 ± 0.52 ^a^	5.60 ± 0.35 ^a^	5.90 ± 0.38 ^a^	5.88 ± 0.05 ^a^	5.78 ± 0.05 ^a^
Total	257.46 ± 2.36 ^a^	268.90 ± 10.34 ^a^	245.78 ± 29.00 ^a^	266.91 ± 8.35 ^a^	269.33 ± 15.61 ^a^
Total phenolic compounds (mg/L of gallic acid equivalents)	1074.79 ± 4.90 ^a^	1039.65 ± 28.27 ^a^	978.42 ± 47.79 ^a^	1075.52 ± 34.87 ^a^	984.21 ± 53.66 ^a^

Results are presented as means ± SD (*n* = 3). Results in the same row with different letters for the same parameter are significantly different (Tukey, *p* < 0.05).

## Data Availability

Data is contained within the article.
